# Mutations of *TP53* and genes related to homologous recombination repair in breast cancer with germline *BRCA1/2* mutations

**DOI:** 10.1186/s40246-022-00447-3

**Published:** 2023-01-06

**Authors:** Jinyong Kim, Kyeonghun Jeong, Hyeji Jun, Kwangsoo Kim, Jeong Mo Bae, Myung Geun Song, Hanbaek Yi, Songyi Park, Go-un Woo, Dae-Won Lee, Tae-Yong Kim, Kyung-Hun Lee, Seock-Ah Im

**Affiliations:** 1 Department of Internal Medicine, Seoul National University College of Medicine, Seoul National University Hospital, 101, Daehak-ro, Jongno-gu, Seoul, 03080 Republic of Korea; 2 Interdisciplinary Program in Bioengineering, Seoul National University, Seoul, Republic of Korea; 3 Transdisciplinary Department of Medicine and Advanced Technology, Seoul National University Hospital, Seoul, Republic of Korea; 4 Department of Pathology, Seoul National University College of Medicine, Seoul National University Hospital, Seoul, Republic of Korea; 5 Biomedical Research Institute, Seoul National University Hospital, Seoul, Republic of Korea; 6 Division of Hematology/Oncology, Department of Internal Medicine, Kangbuk Samsung Hospital, Sungkyunkwan University School of Medicine, Seoul, Republic of Korea; 7 Cancer Research Institute, Seoul National University, Seoul, Republic of Korea

**Keywords:** BRCA, P53, Breast cancer, NGS, Signature 3

## Abstract

**Background:**

Germline mutations of breast cancer susceptibility gene *BRCA1* and *BRCA2* (*gBRCA1/2*) are associated with elevated risk of breast cancer in young women in Asia. BRCA1 and BRCA2 proteins contribute to genomic stability through homologous recombination (HR)-mediated double-strand DNA break repair in cooperation with other HR-related proteins. In this study, we analyzed the targeted sequencing data of Korean breast cancer patients with *gBRCA1/2* mutations to investigate the alterations in HR-related genes and their clinical implications.

**Materials and methods:**

Data of the breast cancer patients with pathogenic *gBRCA1/2* mutations and qualified targeted next-generation sequencing, SNUH FiRST cancer panel, were analyzed. Single nucleotide polymorphisms, small insertions, and deletions were analyzed with functional annotations using ANNOVAR. HR-related genes were defined as *ABL1, ATM, ATR, BARD1, BRCA1, BRCA2, CDKN1A, CDKN2A, CHEK1, CHEK2, FANCA, FANCD2, FANCG, FANCI, FANCL, KDR, MUTYH, PALB2, POLE, POLQ, RAD50, RAD51, RAD51D, RAD54L,* and *TP53*. Mismatch-repair genes were *MLH1, MSH2*, and *MSH6*. Clinical data were analyzed with cox proportional hazard models and survival analyses.

**Results:**

Fifty-five Korean breast cancer patients with known *gBRCA1/2* mutations and qualified targeted NGS data were analyzed. Ethnically distinct mutations in *gBRCA1/2* genes were noted, with higher frequencies of Val1833Ser (14.8%), Glu1210Arg (11.1%), and Tyr130Ter (11.1%) in *gBRCA1* and Arg2494Ter (25.0%) and Lys467Ter (14.3%) in *gBRCA2.* Considering subtypes, *gBRCA1* mutations were associated with triple-negative breast cancers (TNBC), while *gBRCA2* mutations were more likely hormone receptor-positive breast cancers. At least one missense mutation of HR-related genes was observed in 44 cases (80.0%). The most frequently co-mutated gene was *TP53* (38.1%). In patients with *gBRCA1/2* mutations, however, genetic variations of *TP53* occurred in locations different from the known hotspots of those with sporadic breast cancers. The patients with both *gBRCA1/2* and *TP53* mutations were more likely to have TNBC, high Ki-67 values, and increased genetic mutations, especially of HR-related genes. Survival benefit was observed in the *TP53* mutants of patients with *gBRCA2* mutations, compared to those with *TP53* wild types.

**Conclusion:**

Our study showed genetic heterogeneity of breast cancer patients with *gBRCA1* and *gBRCA2* mutations in the Korean populations. Further studies on precision medicine are needed for tailored treatments of patients with genetic diversity among different ethnic groups.

**Supplementary Information:**

The online version contains supplementary material available at 10.1186/s40246-022-00447-3.

## Introduction

Germline mutations of breast cancer susceptibility gene *BRCA1* and *BRCA2* (*gBRCA1/2*) are associated with an elevated lifetime risk of cancer in multiple organs including breast, ovary, colon, prostate, and pancreas [1–3]. Breast cancer in patients with *gBRCA1/2* mutations accounts for 1–4% of all breast cancer, but the prevalence increases up to 8–30% in familial or early-onset breast cancer [4–7]. Especially in the Asian populations, *gBRCA1/2*-associated breast cancer are known to develop in younger age, with higher incidence of germline *BRCA2* (*gBRCA2*) mutation than germline *BRCA1* (*gBRCA1*) when compared to other ethnicities [6, 8, 9].

BRCA1 and BRCA2 proteins act as tumor suppressors that has distinct role in homologous recombination (HR)-mediated double-strand DNA break repair (DDR) [10, 11]. In response to DNA damage, BRCA1 and BRCA2 proteins interact with a numbers of other proteins including BARD1, PALB2, and RAD51 to maintain genomic integrity [12–16]. Tumors with deficiency or mutations in these genes, known as homologous recombination deficiency (HRD), are considered sensitive to poly(adenosine diphosphate [ADP]–ribose) polymerase (PARP) inhibitors that stall replication fork and lead to synthetic lethality [17].

HRD is identified as a potential prognostic and predictive biomarker across multiple cancer types [18–21], and currently, multiple methods to measure HRD are developed [22–24]. While these methods utilizing whole-genome and whole-exome sequencing techniques are expected to harbor in-depth information about the HR-related gene mutations, its application is still expensive, laborious, and time-consuming for most of the patients in clinical settings [25]. Alternatively, targeted sequencing provides genetic mutations and is useful to identify therapeutic biomarkers. In this study, we analyzed the targeted sequencing data of Korean breast cancer patients with *gBRCA1/2* mutations to investigate the alterations in HR-related genes and their clinical implications.

## Materials and methods

### Study design and subjects

This is a retrospective cohort study of the breast cancer patients with pathogenic *gBRCA1/2* mutations from October 2015 to December 2020 at Seoul National University Hospital, Seoul, Korea. The patients with breast cancer of age 20 years or older who had *gBRCA1/2* mutation and SNUH FiRST cancer panel, a targeted next-generation sequencing (NGS) platform, were included. Pathologic diagnosis and immunohistochemistry (IHC) on estrogen receptor (ER), progesterone receptor (PR), and human epidermal growth factor receptor 2 (HER2) status were confirmed by the pathologic reports of surgical and percutaneous biopsies. ER or PR ≥ 1% in IHC were considered hormone receptor-positive. HER2-positive was defined according to the manufacturer’s criteria and ASCO/CAP 2018 guideline.

### Genetic sequencing

Genomic DNA was isolated from peripheral blood leukocytes and were tested for pathogenic *gBRCA1/2* mutation by direct Sanger sequencing. Patients with equivocal variants and variants of unknown significance were excluded from analyses.

DNA was extracted from breast cancer and was subjected to targeted NGS platform named SNUH FiRST Cancer Panel. SNUH FiRST panel included 214 genes (version 3.0), 215 genes (version 3.1), and 216 genes (version 3.2) including microsatellite status with five microsatellite markers (D2S123, D5S346, D17S250, BAT25, and BAT26).

The NGS data with either mean coverage were less than 100X or the proportion of bases with coverage above 50X were less than 80% were regarded as disqualified and were excluded. Adaptor sequences and low-quality reads of targeted sequencing data were trimmed with *fastp* [26]. Trimmed reads were aligned with reference genome UCSC *hg19*, using BWA (version 0.7.17) [27]. Preprocessing was performed using *MarkDuplicates*, *BaseRecalibrator*, and *ApplyBQSR* function of *GATK best practices* (version 4.1.7.0) [28, 29]. After calling single nucleotide polymorphisms (SNP) and small insertions and deletions (INDEL) using Tumor-only mode *Mutect2* in the preprocessed BAM file, the GATK *FilterMutectCalls* function was performed. For both SNP and INDEL, variants satisfying allele depth ≥ 3, total depth ≥ 10, minimum allele depth in both strands > 1, and variable allele frequency (VAF) > 0.2 remained. The strand bias test was conducted by using a Fisher’s exact test, leaving only variants that met the criteria of *p* value > 1 × 10^–6^ for SNP and *p* value > 1 × 10^–20^ for INDEL. Functional annotation of the variants was performed using *ANNOVAR* (version 20,191,024) [30]. Among variants located within the exon or splicing region, variants satisfying minor allele frequency (MAF) ≤ 0.01 in the population databases (Exome aggregation consortium—East Asian, 1000 Genomes project—East Asian, gnomAD—East Asian, NHLBI ESP6500) were used in the downstream analysis [31–34]. Among single nucleotide polymorphisms, silent mutations were excluded. The *TP53* Database R20, July 2019 version was used [35]. R package *maftools* was used to draw the heatmap of HR-related gene mutations and lollipop plots for TP53 amino acid changes [36].

Following list of genes were considered as HR-related genes: *ABL1, ATM, ATR, BARD1, BRCA1, BRCA2, CDKN1A, CDKN2A, CHEK1, CHEK2, FANCA, FANCD2, FANCG, FANCI, FANCL, KDR, MUTYH, PALB2, POLE, POLQ, RAD50, RAD51, RAD51D, RAD54L,* and *TP53* [11, 37]. Mismatch-repair (MMR) genes were defined as *MLH1, MSH2*, and *MSH6*.

### Statistical analyses

Categorical variables were summarized with the frequencies in number and rates in percentages. Continuous variables were represented with the median values and ranges. Differences were assessed using Mann–Whitney test for continuous variables and Pearson’s *χ*^2^ or Fisher’s exact test for categorical variables. Multivariable Cox-proportional hazard models were constructed to find risk factors for survivals and correlation among covariates.

Overall survival (OS) was defined as the time from diagnosis of breast cancer to death of any cause. The OS curves were estimated using the Kaplan–Meier method. If patients survived without death, the survival was censored at the latest date of follow-up when no death was confirmed. Log-rank test *p* value < 0.05 was considered statistically significant. Data and statistical analyses were performed in R version 4.1.3 and RStudio version 2022.02.0 with R packages *survminer* and *survival* [38].

## Results

### Patient characteristics

**Fig. 1:**
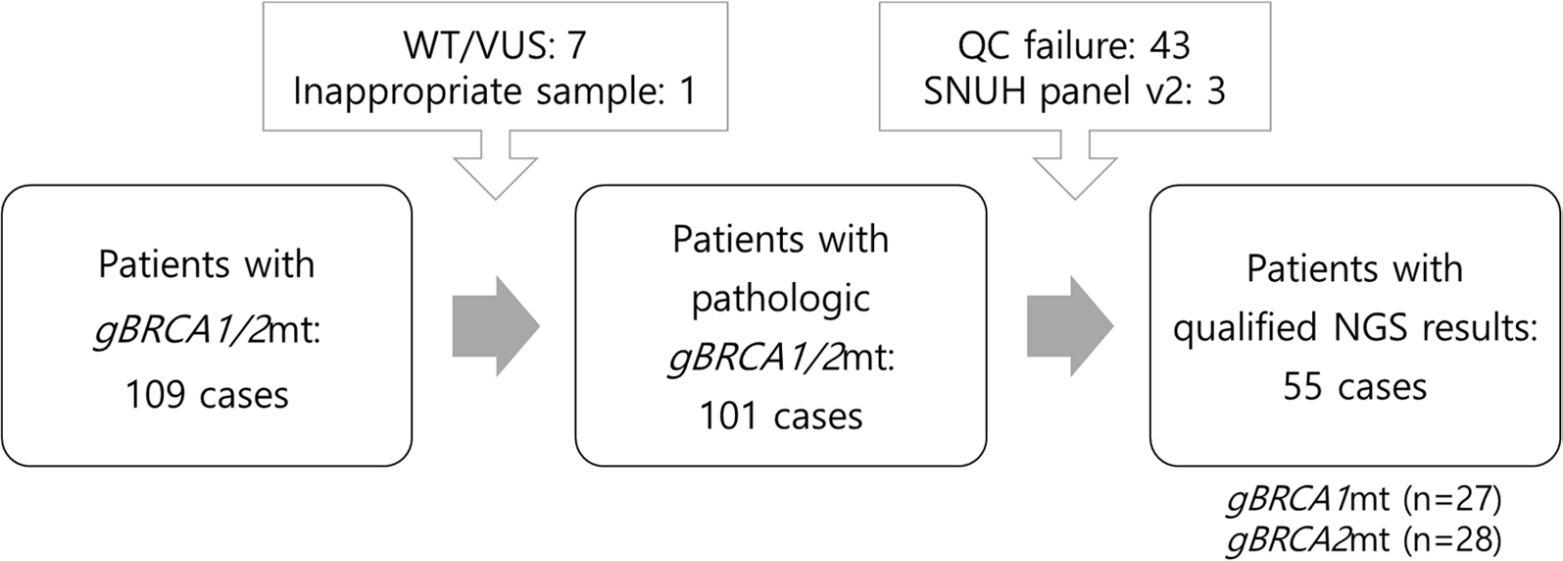
Enrollment of the breast cancer patients with pathogenic *gBRCA1/2* mutations

Among 109 breast cancer patients with known *gBRCA1/2* mutations and targeted NGS data, 7 patients who had silent mutations or variance of unknown significance were excluded. One patient was excluded because NGS was done from uterine adenosarcoma. Forty-six cases failed in quality assurance of NGS. Eventually, 55 patients with pathogenic *gBRCA1/2* mutations with qualified NGS data were included in the study (Fig. 1).

All patients were of Korean ethnicity, and one male patient was included. The median age of all patients at the diagnosis of breast cancer was 42 years old, and majority of the patients were in premenopausal or perimenopausal state (39 of 55, 70.9%). Family history of breast cancer was found in 34 patients (61.8%). Thirteen patients (23.6%) had bilateral breast cancer, and 4 patients (7.3%) also suffered from ovarian cancer. Half of the patients (28 of 55, 50.9%) underwent prophylactic bilateral salpingo-oophorectomy, and 8 patients (14.5%) had prophylactic mastectomy. Most patients (50 of 55, 90.9%) had early breast cancer, and five *de novo* stage IV patients were included. The most common subtype was hormone receptor-positive, HER2-negative breast cancer (28 of 55, 50.9%), followed by triple-negative breast cancer (TNBC, 23 of 55, 41.8%), and hormone receptor-positive, HER2-positive breast cancer (2 of 55, 3.6%). Median Ki-67 value was 10.0. The baseline characteristics are shown in Table 1.

**Table 1:** Patient characteristics

Median (range)	All patients	*gBRCA1*			*gBRCA2*			*p* value (*gBRCA1* vs. *gBRCA2*)
		*TP53* wild type (*N* = 15)	*TP53* mutant (*N* = 12)	*p* value	*TP53* wild type (*N* = 19)	*TP53* mutant (*N* = 9)	*p* value	
Sex								
Female	54 (98.2%)	15 (100.0%)	12 (100.0%)	1.000	18 (94.7%)	9 (100.0%)	1.000	1.000
Male	1 (1.8%)				1 (5.3%)	0 (0.0%)		
Age at diagnosis	42.0 (27–71)	45.0 (29–67)	39.5 (28–55)	0.406	39.0 (27–57)	45.0 (27–71)	0.941	0.980
Family history	34 (61.8%)	10 (66.7%)	7 (58.3%)	0.964	9 (47.4%)	8 (88.9%)	0.092	1.000
Bilateral breast cancer	13 (23.6%)	4 (26.7%)	2 (16.7%)	0.877	2 (10.5%)	5 (55.6%)	0.035	1.000
Ovarian cancer	4 (7.3%)	2 (13.3%)	1 (8.3%)	1.000	1 (5.3%)	0 (0.0%)	1.000	0.577
Menopausal state				0.044			0.534	0.404
Pre- or perimenopausal	39 (70.9%)	9 (60.0%)	12 (100.0%)		13 (68.4%)	5 (55.6%)		
Postmenopausal	15 (27.3%)	6 (40.0%)	0 (0.0%)		5 (26.3%)	4 (44.4%)		
Male	1 (1.8%)				1 (5.3%)	0 (0.0%)		
Prophylactic BSO	28 (50.9%)	8 (53.3%)	6 (50.0%)	1.000	11 (57.9%)	3 (33.3%)	0.418	1.000
Prophylactic mastectomy	8 (14.5%)	4 (26.7%)	2 (16.7%)	0.877	1 (5.3%)	1 (11.1%)	1.000	0.229
TNM stage at diagnosis				0.921			0.080	0.192
I	6 (10.9%)	1 (6.7%)	1 (8.3%)		1 (5.3%)	3 (33.3%)		
II	28 (50.9%)	7 (46.7%)	4 (33.3%)		11 (57.9%)	6 (66.7%)		
III	16 (29.1%)	5 (33.3%)	5 (41.7%)		6 (31.6%)	0 (0.0%)		
IV	5 (9.1%)	2 (13.3%)	2 (16.7%)		1 (5.3%)	0 (0.0%)		
Node positive	37 (67.3%)	10 (66.7%)	9 (75.0%)	0.962	16 (84.2%)	2 (22.2%)	0.006	0.847
Subtypes				0.218			0.185	0.006
Hormone receptor + HER2-	28 (50.9%)	7 (46.6%)	2 (16.7%)		11 (78.9%)	4 (44.4%)		
Hormone receptor + HER2 +	2 (3.6%)	0 ( 0.0%)	0 ( 0.0%)		1 ( 5.3%)	1 (11.1%)		
TNBC	25 (45.5%)	8 (53.3%)	10 (83.3%)		3 (15.8%)	4 (44.4%)		
Ki-67	10.0 (1–90)	10.0 (1–70)	40.0 (10–90)	0.006	5.0 (1–30)	10.0 (2–20)	0.120	0.036
Relapse	23 (41.8%)	6 (40.0%)	3 (25.0%)	0.681	8 (42.1%)	6 (66.7%)	0.418	0.327
Local	3 (5.5%)	1 (6.7%)	0 (0.0%)		2 (10.5%)	0 (0.0%)		
Contralateral	11 (20.0%)	2 (13.3%)	2 (16.7%)		2 (10.5%)	5 (55.6%)		
Distant metastasis	16 (29.1%)	3 (25.0%)	5 (33.3%)		1 (11.1%)	7 (36.8%)		
Death	7 (12.7%)	1 (6.7%)	1 (8.3%)	1.000	5 (26.3%)	0 (0.0%)	0.242	0.449

*gBRCA1* germline *BRCA1*,* gBRCA2* germline *BRCA2*, *BSO* bilateral salpingo-oophorectomy

### Pathogenic gBRCA1/2 mutation

There were 27 patients with pathogenic mutations in *gBRCA1*, and 28 patients with deleterious *gBRCA2* mutations. The only male patient had nonsense mutation in *gBRCA2* (p.Ile332PhefsTer17). While TNBC (*n* = 18, 66.7%) was significantly dominant in the patients with pathogenic *gBRCA1* mutations, hormone receptor-positive breast cancer accounted for the 75% of the patients with pathogenic *gBRCA2* mutations. In the *gBRCA1* mutation group, patients had more TNBC compared to those in *gBRCA2* mutation group (66.7% vs. 25.0%, *p* = 0.006). Median age, family history of breast cancer, prevalence of bilateral breast cancer were similar in both groups (Table 1).

In our study, 27 patients with pathogenic *gBRCA1* mutations and 28 patients with *gBRCA2* mutations were included. The most common variant was Val1833Ser (4 of 27, 14.8%), Glu1210Arg (3 of 27, 11.1%), and Tyr130Ter (3 of 27, 11.1%). Leu1780Pro, Lys307Ser, Trp1815Ter were also found in 7.4% of the patients, respectively. Among *gBRCA2* mutations, Arg2494Ter (7 of 28, 25.0%) and Lys467Ter (4 of 28, 14.3%) were the most common. All mutations found in the patients are listed in Additional file 1: Table 1.

Of the 50 patients who were initially diagnosed as stage I-III, 23 patients (46.0%) experienced relapse. Three patients (6.0%) experienced local recurrence, and eleven patients (22.0%) suffered from recurrent or de novo early breast cancer in contralateral side. Two patients experienced distant metastases after local relapse, and eventually, eleven patients (22.0%) had distant metastases. Death occurred in 7 patients (12.7%). There was no difference of local or distant relapse rates between *gBRCA1* and *gBRCA2* mutants. Relapse-free survival of the stage I–III patients was not different between those with *gBRCA1* and *gBRCA2* mutations (median RFS 138 months vs. 112 months, *p* = 0.89). Overall survival of the patients with *gBRCA1* mutation was also not significantly different from those with *gBRCA2* mutation (median OS 290 months vs. not reached, *p* = 0.41) (Additional file 1: Fig. 1).

### Targeted NGS and HR-related genes

Tissues were obtained from primary breast lesions, lymph nodes, lung, liver, and soft tissue for the targeted NGS. Most of the samples (45 of 55, 81.8%) were obtained from the breast primary lesion. About half (26 of 55, 47.3%) were obtained after lines of chemotherapy treatments. Median tumor proportion was 70%.

In the targeted NGS of 55 patients, 348 mutations were observed: 269 nonsynonymous single nucleotide variations (SNV), 30 nonsense mutations, 12 non-frameshift insertion or deletion, 4 non-frameshift substitutions, 25 frameshift insertions or deletions, and 8 splicings. There were 29 somatic *BRCA2* mutations and 26 somatic *BRCA1* mutations, including 3 cases without detected mutations in either *BRCA1* or *BRCA2* and 3 cases with mutations in both genes.

**Fig. 2:**
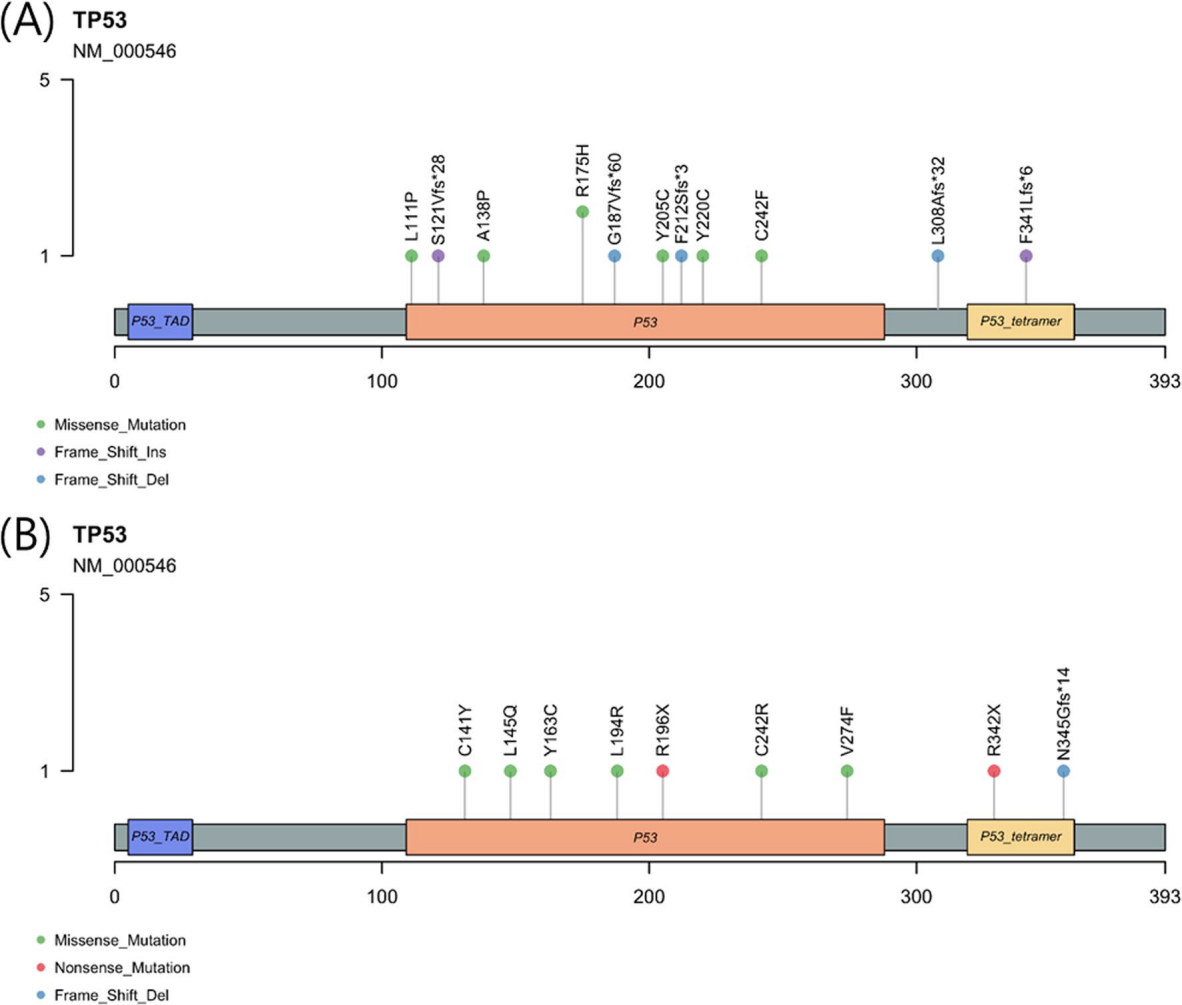
*TP53* mutations in **A** *gBRCA1* mutants and **B** *gBRCA2* mutants

The most frequently co-mutated gene was *TP53* (21 of 55, 38.1%). In the NGS analysis, mutations in *TP53* gene included 6 frameshift insertion or deletion, 2 truncating mutations, and 13 nonsynonymous SNVs. Nonsynonymous SNVs mainly occurred in DNA-binding domain, while frameshift indels and stopgains occurred in oligomerization domain which interacts with other HR-related genes. Among the missense SNVs, five codons were located at DNA-binding grooves and two were at zinc binding sites (Fig. 2).

**Fig. 3:**
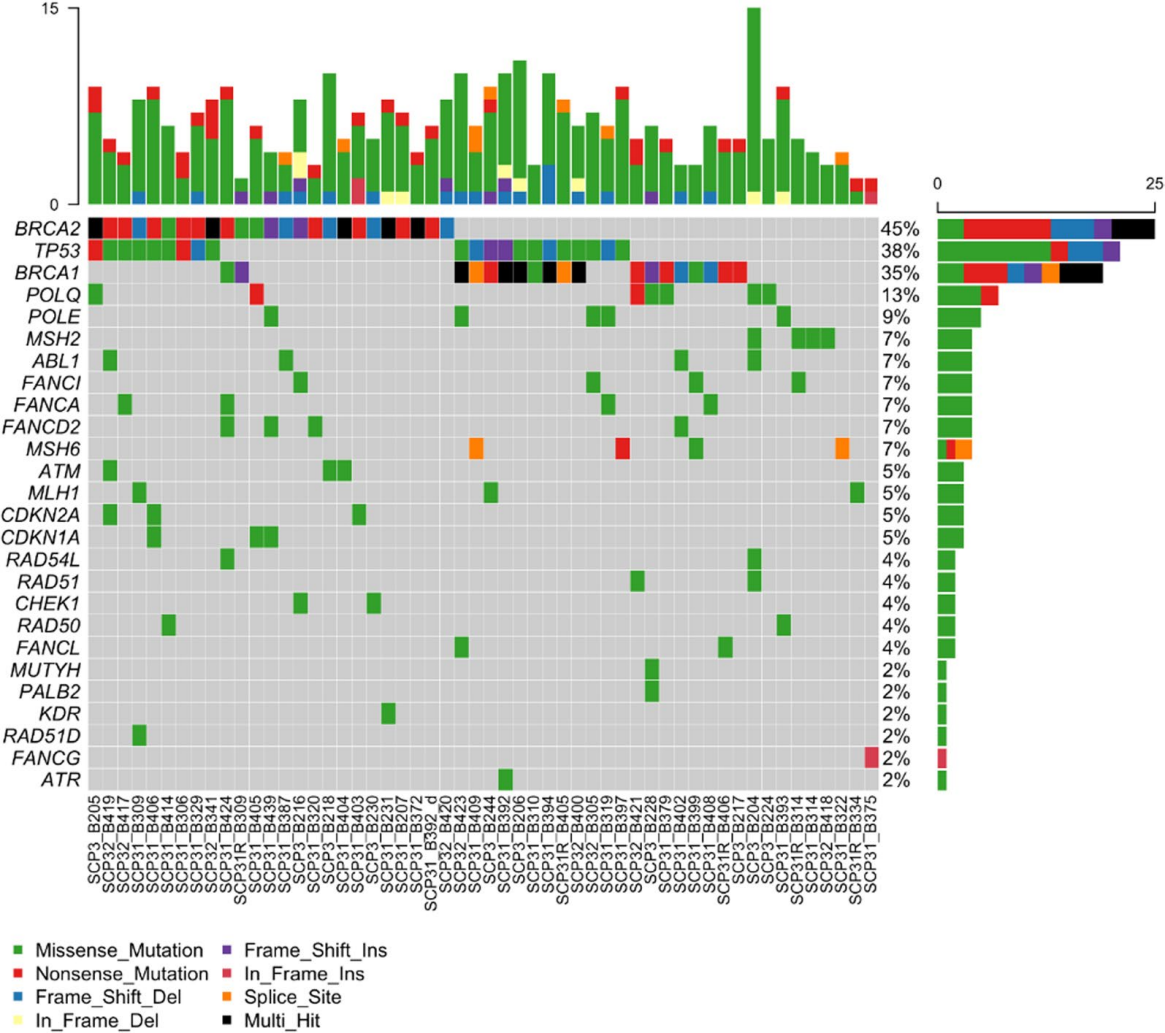
Heatmap of HR-related gene mutations

Among the HR-related genes, the most frequently mutated genes following *TP53* were POLE (7 of 55, 12.7%), ABL1 (4 of 55, 7.3%), and FA-related genes, including FANCA, FANCD2, and FANCI (4 of 55, 7.3%, respectively). ATM was found exclusively in 3 patients with *gBRCA2. PALB2* was observed in one patient with *gBRCA1* mutation. At least one missense mutation in HR-related genes was observed in 44 cases (80.0%). One patient with *gBRCA1* mutation had somatic mutation in BER-related gene, *MUTYH*. Somatic mutations in the mismatch-repair genes (MLH1, MSH2, MSH6) were also observed in 5–7% of the patients with *gBRCA1/2* mutations (Fig. 3).

**Fig. 4:**
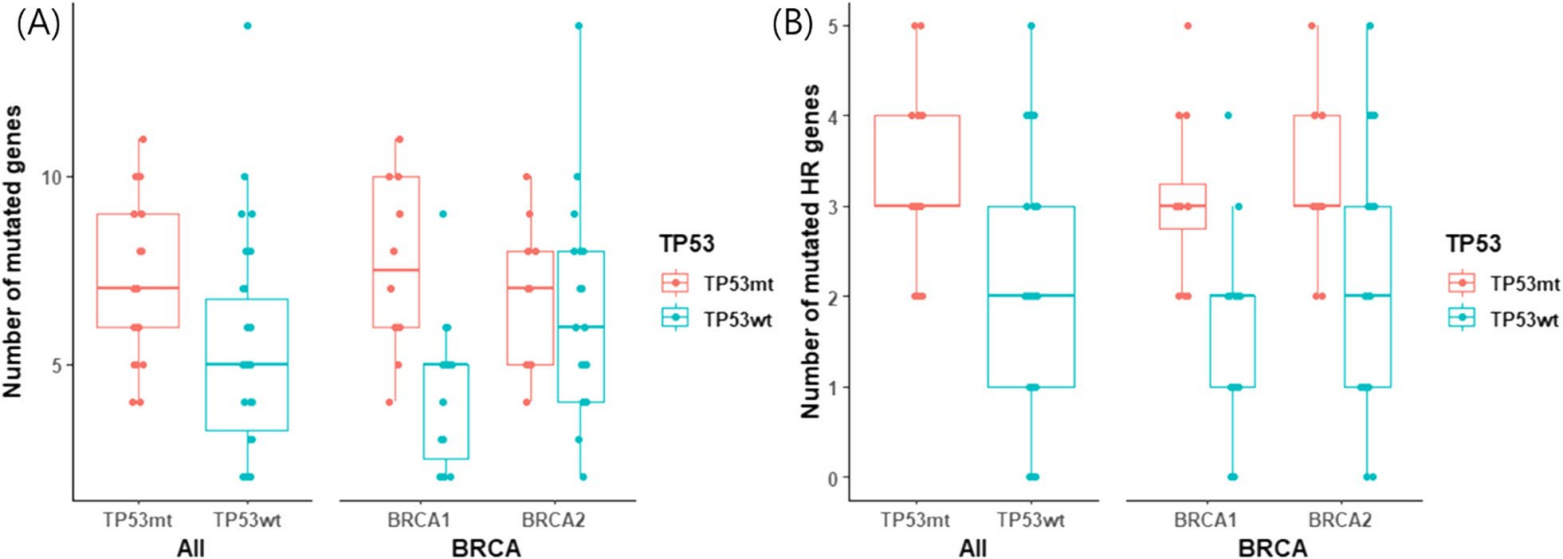
Number of mutated genes by *TP53* mutation status **A** of all genes and **B** of HR-related genes

A total number of mutated genes were significantly higher in the tumors with *TP53* mutations (mean 7.38 vs. 5.35, *p* = 0.003) (Fig. 4). In further analysis, only the number of the mutations in HR-related genes was significantly different (3.14 vs. 1.94, *p* < 0.001), but not that of the non-HR-related genes (4.24 vs. 3.41, *p* = 0.135) (Fig. 4, Additional file 1: Fig. 2). MMR genes were also not affected by the TP53 mutations (0.190 vs. 0.206, *p* = 0.901). Both *gBRCA1*- and *gBRCA2*-mutated tumors showed higher prevalence HR-related gene mutations in *TP53* mutants compared to *TP53* wild types (3.08 vs. 1.67, *p* = 0.003 in *gBRCA1*, 3.22 vs. 2.16, *p* = 0.041 in *gBRCA2*, respectively).

### Clinical significance of TP53 co-mutation

The patients who had both *TP53* mutation and *gBRCA1/2* mutation were significantly associated with TNBC (*p* = 0.028) and higher Ki-67 (*p* = 0.001). Among the patients who had *gBRCA1,* the median age at the diagnosis of breast cancer was 39.5 years in those with concurrent mutation in *TP53*, compared to 45 years without *TP53*. The patients also had significantly more premenopausal status and higher Ki-67 values (*p* = 0.044, 0.006, respectively). Contrastingly, in the patients who had *gBRCA2* and TP53 co-mutations, the median age was 45 years compared to 39 years without mutations in *TP53*. In these patients, the incidence of bilateral breast cancer and node-negative diseases was significantly higher than that with wild type *TP53* (*p* = 0.035 and 0.006, respectively) (Table 1).

**Fig. 5:**
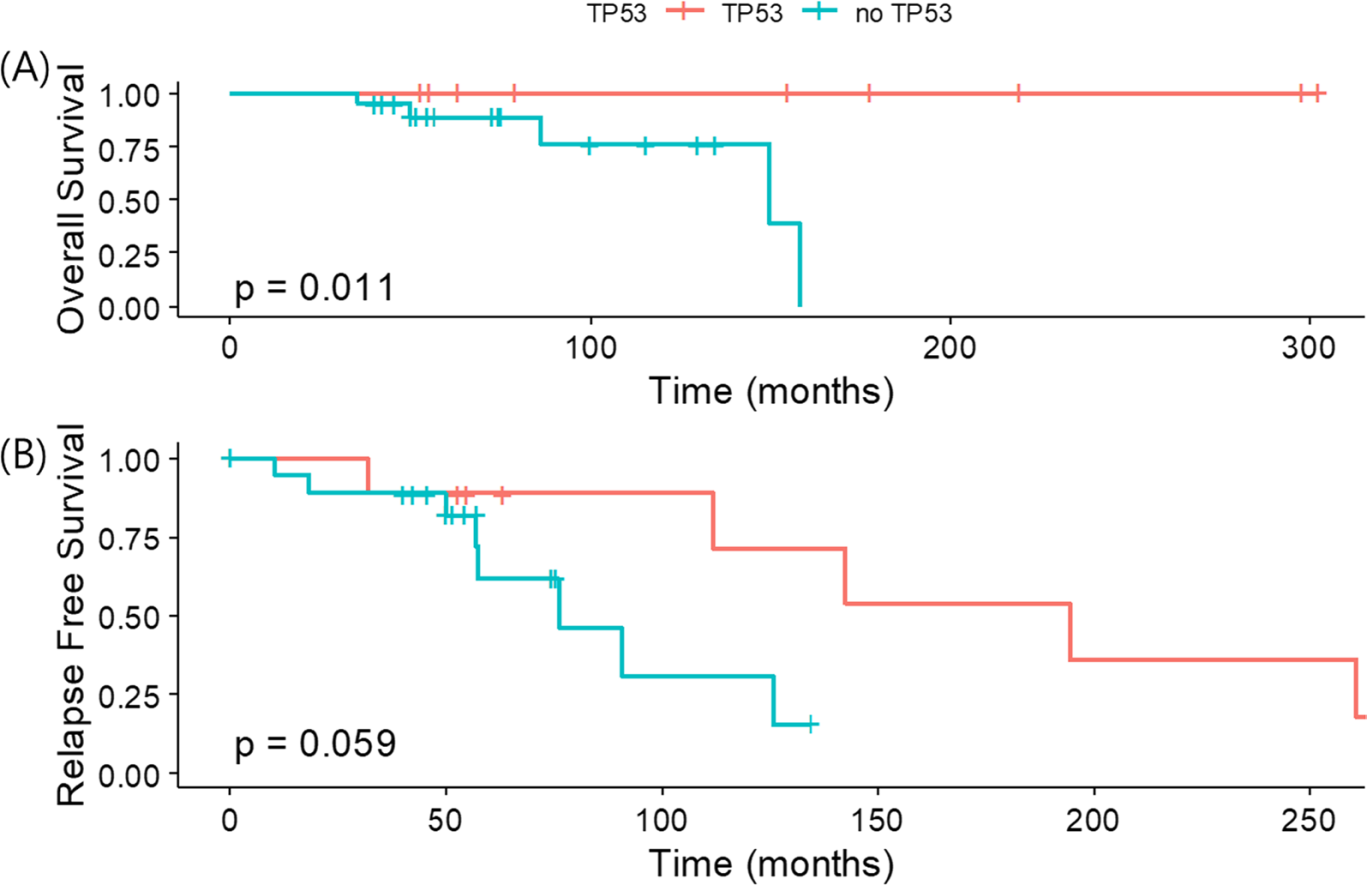
Survivals of patients with *gBRCA2*mt by *TP53* mutation status **A** overall survival and **B** relapse-free survival

Interestingly, these *gBRCA2*-related patients with *TP53* co-mutation also showed superior overall survival to those without *TP53* mutations (*p* = 0.011) (Fig. 5). After the exclusion of de novo stage IV breast cancer in the *gBRCA2* group, the relapse-free survival was numerically longer in those with *TP53* co-mutation compared to *TP53* wild types. Mutation status of *TP53* did not affect the survival of patients with *gBRCA1* mutations (Additional file 1: Fig. 3). Unfortunately, due to small number of deaths and total cases, none of other factors including age, family history of breast cancer, TNM staging, PR-positivity, or Ki-67 was significant in Cox-proportional hazard models (data not shown).

## Discussion

Clinical and genetic characteristics of breast cancer patients with pathogenic *gBRCA1/2* mutations were investigated in the present study. In consistence with previous reports, about half of our patients were diagnosed before age 40, and majority were premenopausal [4, 39]. These patients also presented with other high risk features, such as family history of breast cancer, co-occurrence of contralateral breast or ovarian cancer, and high Ki-67 index [5, 7, 40]. Carriers of *gBRCA1* mutations had higher prevalence of triple-negative breast cancers (TNBC), while majority of *gBRCA2* mutations were associated with hormone receptor-positive breast cancers [41, 42]. However, TNM staging, histologic grade of the tumor, and lymph node metastases were not statistically different between *gBRCA1*-mutated and *gBRCA2*-mutated patients.

Previously noted *gBRCA1/2* mutations in Korean ethnicity were consistently detected in our patients. The most common mutation of *BRCA2*, p.Arg2494X, was also detected in 12–15% of Koreans with *gBRCA1/2* mutations in other studies and suggested as a founder mutation [43, 44]. Among *BRCA1* variants, p.Val1833Serfs, p.Tyr130Ter, Glu1210Argfs, and p.Trp1815Ter had been frequently observed also in Asian populations [44–46]. Moreover, there were two cases of *BRCA1* p.Leu1780Pro, which was also recently identified as a novel pathogenic variant [47]. None of the known or suspected founder mutations of Ashkenazi Jews [48], Caucasians [49], North African [50], Hispanic [51], or Mexican populations [52] were observed. Interestingly, the Greek founder mutation *BRCA1*, p.Val1833Met and one of our common variants p.Val1833Serfs had different amino acid change in same location [53]. These results highlight the ethnical differences among *gBRCA1/2* mutations.

While BRCA genes are known as the strongest drivers of the breast cancers, 35 of the 93 known driver mutations were also observed in these patients [54]. The most frequently co-mutated gene was *TP53*, which usually undergo missense mutations in DNA-binding domains and nonsense or deletions in other domains [40, 55]. In our study, only one-third of the *TP53* mutations were found in major grooves or zinc binding site of the DNA-binding domain [56]. Of all, only ten cases were included in 73 codon hotspots defined by Walker et al. [57, 58], and two cases of *TP53* p.R175H were observed in our patients among six well-known hotspot codons (R175, R213, G245, R248, R273, and R282) that account for a quarter of all *TP53* mutations. All in all, the codon distribution and types of *TP53* mutations of *gBRCA1/2* mutants had discrepancy from those in known hotspots of sporadic breast cancers [59].

*TP53* acts as a tumor suppressor gene, and its mutations were strongly associated with increased chromosomal instability and higher HRD score [60]. In patients with *TP53* mutations in addition to *gBRCA1/2* mutations, the total number of mutated genes, especially of HR-related genes, increased. In *gBRCA1* mutants, mutation of non-HR-related genes also increased, probably due to its tendency toward more frequent structural rearrangements than *gBRCA2* [61].

*TP53* mutations are associated with breast cancer with younger age, higher grade, advanced stages, hormone receptor negativity, enrichment of mutational signature 3, and with a high HRD index [54, 62]. In our study, the tumors with *TP53* mutation were also more likely to be TNBC and have high Ki-67 values. On the other hand, age and staging at initial diagnosis of the patients with *TP53* mutations were not significantly different from those with *TP53* wild types. High rate of *TP53* mutations observed from younger ages and early stages of the patients with *gBRCA1/2* mutations implied the effect of DNA-repair deficiency and its selective pressure on tumor suppressor genes [57, 63].

The role of *TP53* variants in the breast patients with pathogenic *gBRCA1/2* mutation had been controversial. In our study, the improvement of overall survival was observed only in the patients with *gBRCA2* mutations, despite the similar pathological complete remission rates after neoadjuvant chemotherapies, relapse rates, and de novo stage IV diseases. Those with wild-type *TP53* and *gBRCA2* mutation had numerically higher rate of distant metastases and deaths. As for subtypes of breast cancer, *TP53* mutation has been reported to be more prevalent in basal-like subtypes [63–65]. Consistently, tumors with *TP53* mutants (44.4%) were more likely to be ER-negative than those with wild-type *TP53* (15.8%) in the present study. Previous study showed that, Asians were more likely to have *TP53* mutations among ER-positive breast cancers than Caucasians, and *TP53* mutations were associated with poor survival in ER-positive breast cancer [63]. Another study has shown that the neoadjuvant chemotherapies, regardless of the pathological complete remission rates, were more effective in the patients with *TP53* mutations than wild types [66]. Taken together, one possible explanation is that initially luminal-like *gBRCA2*-mutated breast cancer are affected by *TP53* co-mutation to become closer to basal-like entities, and more sensitive to neoadjuvant and adjuvant chemotherapy that led to less distant metastases and deaths.

Our study has limitations that arise from the retrospective design utilizing the targeted NGS for clinical purpose. Genetic mutations not targeted in the panels were difficult to be observed, including large deletions, rearrangements, chromosomal abnormalities, and methylations. Moreover, targeted NGS was done for clinical purposes and the paired biopsy with non-neoplastic tissues was difficult to be done. Small number of the patients due to the rarity of *gBRCA1/2* mutations were another limitations in obtaining statistical significance. Still, our study holds its value in delineating the rare breast cancer entity with *gBRCA1/2* and concomitant somatic mutations, especially in the Asian populations.

## Conclusion

Our study showed genetic heterogeneity of pathogenic mutations in *gBRCA1* and *gBRCA2* in the Korean populations. Patterns of *TP53* mutations in concomitant *gBRCA1/2* mutations were distinct from those in sporadic breast cancers. Co-mutation of *gBRCA1/2* and *TP53* genes was associated with TNBC, high Ki-67, and higher number of mutated genes related to HR pathways. Further studies are needed to clarify the association between genetic diversity among different ethnic groups and clinical circumstances to develop treatment strategies that could lead to better survivals.

## Supplementary Information

**Additional file 1.  Figure S1**: Survivals of patients with gBRCA1mt and gBRCA2mt (A) overall survival and (B) relapse-free survival. ** Figure S2**: Number of mutated non-HR-related genes by TP53 mutation status. **Figure S3**: Survivals of gBRCA1 patients by TP53 mutation status (A) overall survival and (B) relapse-free survival.** Table S1**: Germline BRCA1/2 mutations

## Data Availability

The datasets used and/or analyzed during the current study are available from the corresponding author on reasonable request.
