# Supplementary material for: Mutations of TP53 and genes related to homologous recombination repair in breast cancer with germline BRCA1/2 mutations
Source: Hum Genomics. 2023 Jan 6;17:2. doi: 10.1186/s40246-022-00447-3 (PMC9817339; doi:10.1186/s40246-022-00447-3)

| *gBRCA1* (N=27) | | | *gBRCA2* (N=28) | | |
| --- | --- | --- | --- | --- | --- |
| Nucleotide change | Protein change | Frequncy | Nucleotide change | Protein change | Frequncy |
| 5496_5506delinsA | Val1833Serfs | 4 (14.8%) | 7480C>T | Arg2494Ter | 7 (25.0%) |
| 3627dupA | Glu1210Argfs | 3 (11.1%) | 1399A>T | Lys467Ter | 3 (10.7%) |
| 390C>A | Tyr130Ter | 3 (11.1%) | 6553del | Ala2185fs | 1 (3.6%) |
| 5339T>C | Leu1780Pro | 2 (7.4%) | 5471del | Asn1842Metfs | 1 (3.6%) |
| 911_918dupTCTGTAAT | Lys307Ser | 2 (7.4%) | 5656C>T | Gln1886Ter | 1 (3.6%) |
| 5445G>A | Trp1815Ter | 2 (7.4%) | 8140C>T | Gln2714Ter | 1 (3.6%) |
| 5251C>T | Arg1751Ter | 1 (3.7%) | 9076C>T | Gln3026Ter | 1 (3.6%) |
| 332-12A>G | c.213-12A>G | 1 (3.7%) | 97G>T | Glu33Ter | 1 (3.6%) |
| 5074+1G>T | c.5074+1G>T | 1 (3.7%) | 1433>A | Gly402Asp | 1 (3.6%) |
| 5467+1G>A | c.5467+1G>A | 1 (3.7%) | 5576_5579del | Ile1859fs | 1 (3.6%) |
|  | exon 1-14 | 1 (3.7%) | 8023A>G | Ile2675Val | 1 (3.6%) |
| 4981G>T | Glu1661Ter | 1 (3.7%) | 994del | Ile332fs | 1 (3.6%) |
| 5080G>T | Glu1694Ter | 1 (3.7%) | 2041insA | Ile605fs | 1 (3.6%) |
| 1716del | Glu572fs | 1 (3.7%) | 3096_3110delinsT | Lys1032fs | 1 (3.6%) |
| 3347_3348delAG | Gly1077fs | 1 (3.7%) | 8329A>T | Lys2777Ter | 1 (3.6%) |
| 922_924delinsT | Ser308Ter | 1 (3.7%) | 8298_8299dup | Pro2767fs | 1 (3.6%) |
| 2269del | Val757fs | 1 (3.7%) | 3744_3747del | Ser1248fs | 1 (3.6%) |
|  |  |  | 2798_2799del | Thr933fs | 1 (3.6%) |
|  |  |  | 8951C>A | Ser2984Ter | 1 (3.6%) |
|  |  |  | 9431del | Ser3144fs | 1 (3.6%) |

Supplementary Table 1. Germline *BRCA1/2* mutations

Supplementary Figure 1. Survivals of patients with *gBRCA1*mt and *gBRCA2*mt (A) overall survival and (B) relapse-free survival


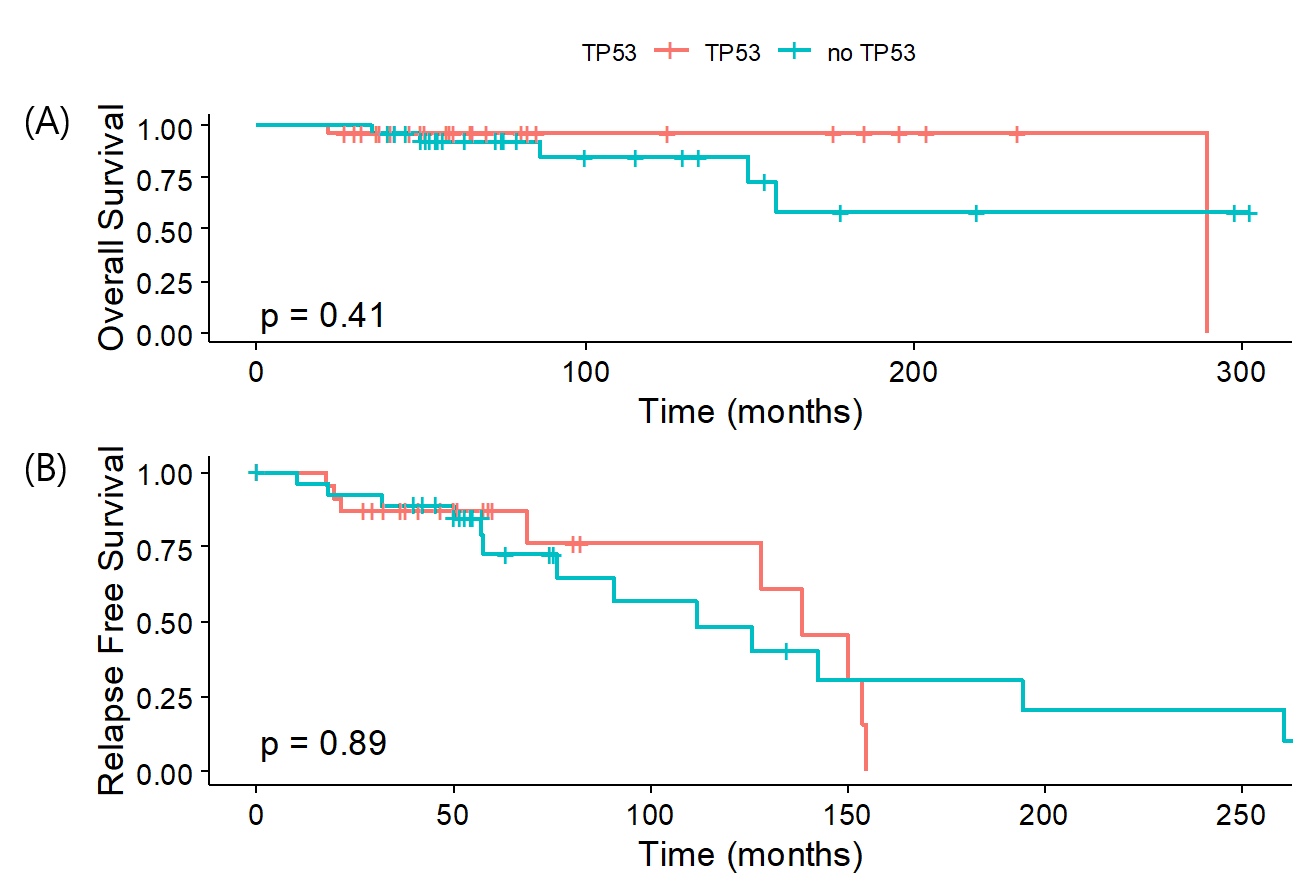


Supplementary Figure 2. Number of mutated non-HR-related genes by *TP53* mutation status


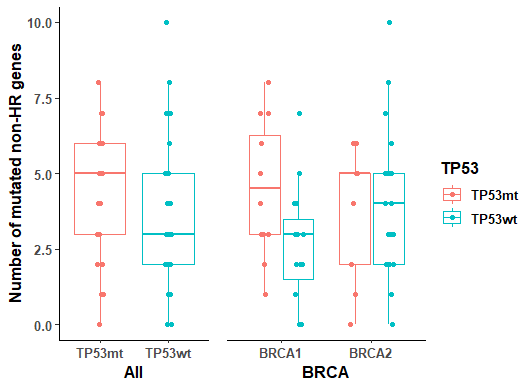


Supplementary Figure 3. Survivals of *gBRCA1* patients by *TP53* mutation status (A) overall survival and (B) relapse-free survival


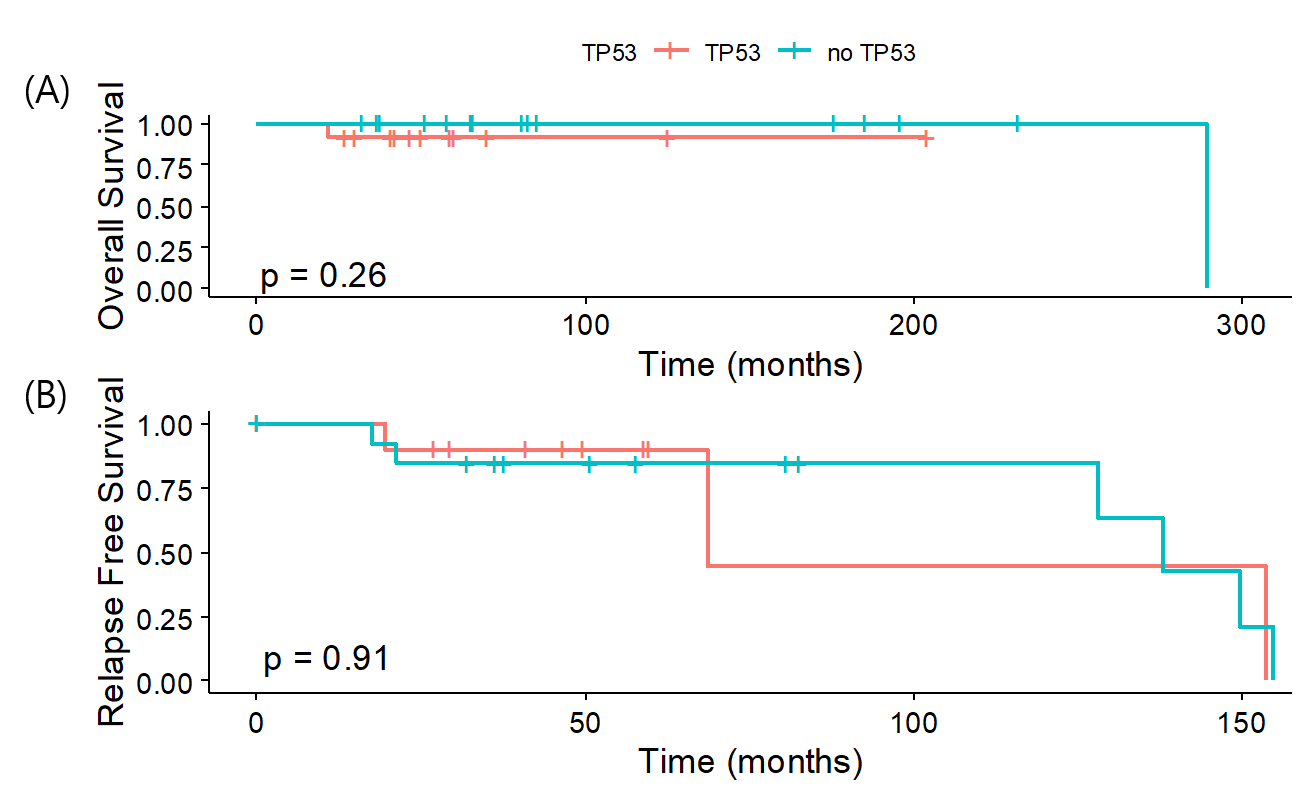

Supplement: Supplementary file 1 — Additional file 1. Figure S1: Survivals of patients with gBRCA1mt and gBRCA2mt (A) overall survival and (B) relapse-free survival. Figure S2: Number of mutated non-HR-related genes by TP53 mutation status. Figure S3: Survivals of gBRCA1 patients by TP53 mutation status (A) overall survival and (B) relapse-free survival. Table S1: Germline BRCA1/2 mutations [file 40246_2022_447_MOESM1_ESM.docx]
